# Thermal Transitions and Structural Characteristics of Poly(3,4-ethylenedioxythiophene/cucurbit[7]uril) Polypseudorotaxane and Polyrotaxane Thin Films

**DOI:** 10.3390/ma17061318

**Published:** 2024-03-13

**Authors:** Barbara Hajduk, Paweł Jarka, Henryk Bednarski, Henryk Janeczek, Pallavi Kumari, Aurica Farcas

**Affiliations:** 1 Centre of Polymer and Carbon Materials, Polish Academy of Sciences, 34 Marie Curie-Skłodowska Str., 41-819 Zabrze, Poland; hbednarski@cmpw-pan.pl (H.B.); hjaneczek@cmpw-pan.pl (H.J.); pkumari@cmpw-pan.pl (P.K.); 2 Department of Engineering Materials and Biomaterials, Silesian University of Technology, 18a Konarskiego Str., 41-100 Gliwice, Poland; pawel.jarka@polsl.pl; 3 “Petru Poni” Institute of Macromolecular Chemistry, Romanian Academy, Grigore Ghica Voda Alley, 41A, 700487 Iasi, Romania

**Keywords:** PEDOT, supramolecular encapsulation, cucurbit[7]uril, variable-temperature spectroscopic ellipsometry, organic semiconductors, thin polymer films, surface morphology

## Abstract

Herein, we report the thermal transitions and structural properties of poly(3,4-ethylenedioxythiophene/cucurbit[7]uril) pseudopolyrotaxane (PEDOT∙CB7-PS) and polyrotaxane (PEDOT∙CB7-PR) thin films compared with those of pristine PEDOT. The structural characteristics were investigated by using variable-temperature spectroscopic ellipsometry (VTSE), differential scanning calorimetry (DSC), X-ray diffraction (XRD) and atomic force microscopy (AFM). VTSE and DSC results indicated the presence of an endothermic process and glass transition in the PEDOT∙CB7-PS and PEDOT∙CB7-PR thin films. X-ray diffraction of PEDOT∙CB7-PS and PEDOT∙CB7-PR powders displayed the presence of interchain π-π stacking revealing a characteristic arrangement of aromatic rings in the internal structure of the crystallites. AFM imaging of PEDOT∙CB7-PS and PEDOT∙CB7-PR thin films exhibited significant differences in the surface topographies compared with those of PEDOT. A high degree of crystallization was clearly visible on the surface of the PEDOT layer, whereas the PEDOT∙CB7-PS and PEDOT∙CB7-PR thin films exhibited more favorable surface parameters. Such significant differences identified in the surface morphology of the investigated layers can, therefore, be clearly associated with the presence of surrounding CB7 on PEDOT skeletons.

## 1. Introduction

**Figure 1:**
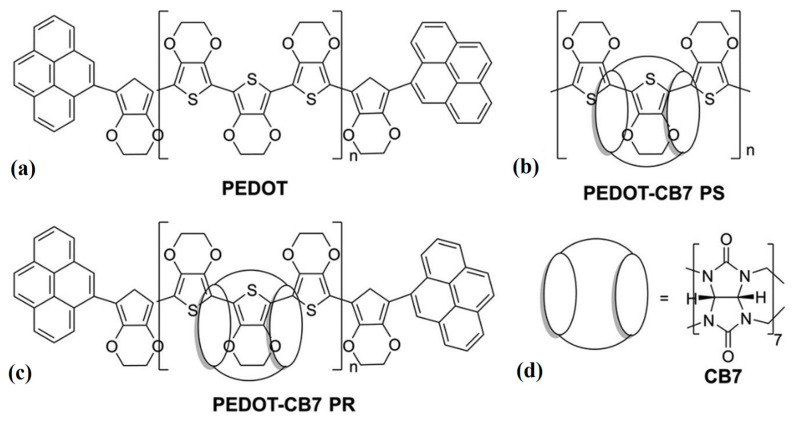
Chemical structures of pristine PEDOT (**a**), PEDOT∙CB7-PS (**b**) PEDOT∙CB7-PR (**c**), and CB7 (**d**).

Poly(3,4-ethylenedioxythiophene) (PEDOT) doped with poly(4-styrene sulfonate) (PSS) is widely recognized as an organic semiconductor material whose photo-physical properties are of great interest for application in various optoelectronic fields [1,2]. Owing to the good hole conductivity and high optical transparency of PEDOT:PSS, its dispersion in water is widely used to produce auxiliary layers in organic devices and hybrid solar cells [3,4,5]. It should be mentioned that in the aqueous PEDOT:PSS dispersions, PEDOT chains form coils that pack randomly in solid-state films, which affect charge-transport properties. The electrical conductivity of PEDOT:PSS layers depends on the content of conductive PEDOT, with PSS playing two important roles as a doping agent and ensuring good dispersion in water. For the latter reason, PSS is always present in excess in aqueous PEDOT:PSS dispersions. The properties of this polyelectrolyte complex have been the subject of intensive research [6,7,8]. There is even a stream of work dedicated to optimizing the conductivity of PEDOT:PSS layers by using various additives to the solution or treating the layers themselves after deposition [9,10,11,12,13,14]. Another direction of research worth mentioning is work on developing non-contact methods for determining the electrical conductivity of layers without placing contacts on them [15,16,17]. For example, Bednarski et al. [18] reported the effect of the PEDOT to PSS ratio on the optical properties of PEDOT:PSS thin films. In this approach, a composition-dependent optical model of PEDOT:PSS layers was developed for spectroscopic ellipsometry in the UV–VIS–NIR spectral range. Moreover, in this work, the optical properties of PEDOT:PSS thin films were successfully related to their electrical conductivity within the framework of the generalized effective medium theory. In this way, it was possible to determine the electrical conductivity of PEDOT:PSS layers based solely on optical measurements, which are clearly non-contact in nature. Despite the great potential of PEDOT, its limited solubility is a major obstacle in applications. Many efforts have been adopted to improve PEDOT’s solubility through molecular design, but its solubility in organic solvents is not optimal either [19]. This is why there is a lot of research on improving the solubility and processability of PEDOT material. In addition, supramolecular chemistry offers a versatile approach to constructing complex structures using non-covalent interactions [20]. Therefore, the construction of PEDOT∙CB7-PS and PEDOT∙CB7-PR, in which PEDOT chains are wrapped around cucurbit[7]uril (CB7), is noteworthy as an alternative approach to improving the solubility and photophysical properties of the PEDOT material. The main goal in the exploration of these new materials was to get rid of the non-conductive part of PEDOT:PSS (i.e., redundant PSS) and work solely with conductive PEDOT complexes. While our recent studies [21,22] demonstrated the favorable effects of PEDOT encapsulation into the CB7 cavity on the solubility, optical, electrochemical, morphological, and electrical properties of PEDOT, the thermal characteristics need further in-depth investigation. Regarding the thermal properties of these complex materials, only the results of thermogravimetric analysis (TGA) and no glass transitions or melting temperatures between 20 and 200 °C were reported. It should be mentioned that the stable dispersions of PEDOT∙CB7-PS and PEDOT∙CB7-PR in water have potential for application as intrinsically luminescent materials [22]. Based on the above discussion and motivated by the new prospects of these PEDOT supramolecular materials with EDOT repeat units enclosed in the CB7 cavities, we decided to select the water–DMSO-soluble fractions of PEDOT∙CB7-PS and PEDOT∙CB7-PR and study their thermal transitions and structural characteristics, which are instrumental for future applications. The chemical structures of the investigated PEDOT, PEDOT∙CB7-PS, and PEDOT∙CB7-PR compounds are shown in Figure 1. Therefore, for the first time, we analyze the thermal transitions occurring in PEDOT CB7-PS and PEDOT CB7-PR thin films. The thermal analysis was performed using VTSE spectroscopy and then correlated using DSC data. Further, by combining XRD analysis of the crystallographic structure, optical studies, and surface morphologies, we expanded our knowledge, and PEDOT CB7-PS and PEDOT CB7-PR thin films were investigated in-depth beyond their previously reported properties [21,22]. The VTSE technique is of great interest due to its high sensitivity to various types of thermal transitions occurring in thin organic layers and good correlation with DSC data [23,24]. Characteristic thermal transition temperatures can be evaluated based on raw ellipsometric data, such as Ψ(T) [25,26,27,28,29,30], Δ(T) [31,32,33,34,35], or their derivatives [36,37,38,39], or derived from the analysis of temperature changes in the course of physical parameter curves, such as thin-layer thickness, refractive index, or electrical resistance [40,41,42,43].

Herein, we report the thermal and morphological properties of PEDOT∙CB7-PS and PEDOT∙CB7-PR thin films spin-coated on glass substrates from (DMSO/H_2_O 1/1 *v*/*v*) solutions using VTSE, DSC, and AFM techniques and compare them with those of pristine PEDOT. The presence of endothermic peaks (*T_end_*) and glass transition temperatures (*T_g_*) for the PEDOT∙CB7-PS and PEDOT∙CB7-PR thin films were confirmed by VTSE and DSC techniques, while cold crystallization was identified only by VTSE. These two complementary techniques indicated that the values of the *T_end_* and *T_g_* temperatures were highly consistent. XRD analysis of PEDOT∙CB7-PS and PEDOT∙CB7-PR powders revealed the presence of interchain π-π stacking. AFM images of the investigated PEDOT∙CB7-PS and PEDOT∙CB7-PR layers exhibited significant differences in the surface topographies compared with that of pristine PEDOT.

## 2. Materials and Methods

### 2.1. Materials

3,4-Ethylenedioxythiophene (EDOT) was purchased from Sigma-Aldrich (Saint Louis, MO, USA) and purified before use by vacuum distillation. Pyrene and anhydrous iron (III) chloride (FeCl_3_) were purchased from Sigma-Aldrich and used as received. Dimethyl sulfoxide (DMSO) and other solvents were of analytical grade (Sigma-Aldrich and Fischer Chemicals AG (Zurich, Switzerland)) and used without further purification.

### 2.2. Characterization

#### 2.2.1. Optical and Thermal Characterization

All the ellipsometric measurements were obtained using a SENTECH SE850E ellipsometer (SENTECH Instruments GmbH, Berlin, Germany), working in a 240–2500 nm spectral range, controlled with SpectraRay 3 software. The ellipsometer was operated in transmission, variable-angle (VASE), and variable-temperature (VTSE) modes. The ellipsometric angles Ψ and Δ were obtained for an incidence angle range of 40–70° at 5° intervals. The VTSE tests were performed using a temperature chamber maintained at a low vacuum (10**^−^**^1^ Torr). The exact temperature settings were applied using the INSTEC mK1000 controller (Instec, Inc., Boulder, CO, USA), which controls the operation of the electric heater and the liquid nitrogen cooling circuit. We used the rapid cooling protocol, in which the value of the maximum heating temperature depends on the type of material being tested. Accordingly, each sample was heated to a temperature close to its melting point for 2 min and then cooled to −50 °C at an average cooling rate of 100 °C∙min^−1^. VTSE examinations of thermal transitions were taken in the wavelength range 250–950 nm. The time intervals between individual measurements were 10 s. Measurements were performed in heating mode at a heating rate equal to 2.0 °C∙min^−1^. However, thermal analysis using differential scanning calorimetry was performed on a Q2000 instrument (TA Instruments, Newcastle, DE, USA) using aluminum sample containers. Measurements were performed in a nitrogen atmosphere (at a gas flow of 50 mL∙min^−1^). High purity indium standards were used to calibrate the instrument. DSC tests were carried out on powder materials from the supplier and those obtained from very thick foils (approximately 2 µm) that were scraped from glass substrates. The cooling and heating rate during measurements was 20 °C∙min^−1^.

#### 2.2.2. Structural Characterization

X-ray diffraction (XRD) studies were performed on a D8 Advance diffractometer (Bruker, Karlsruhe, Germany) equipped with a Cu-Kα cathode (λ = 1.54 Å) using the coupled Two-Theta/Theta mode on polymer powders. The measurement rate was 1.2 °C∙min^−1^ with an angular step of 0.02° ranging from 2° to 60° for 2 theta (dwell time 1 s). Background subtraction from air-particle scattering was performed using DIFFRAC.EVA software V5.1. The surface morphology was examined using a Park Systems XE 100 atomic force microscope operated by the XEI computer program (Suwon, Republic of Korea). XEI enables simultaneous image processing and analysis of the roughness parameters of the tested surface. Microscopic examinations were performed in non-contact mode.

### 2.3. Synthesis and Characterization of PEDOT, PEDOT∙CB7-PS, and PEDOT∙CB7-PR

The synthesis and characterization of PEDOT, PEDOT∙CB7-PS, and PEDOT∙CB7-PR are described in detail elsewhere [21,22]. The coverage ratio of PEDOT∙CB7-PS and PEDOT∙CB7-PR estimated from ^1^H-NMR analysis in D_2_O indicated that almost two EDOT structural units were threaded by one CB7 macrocycle [21,22]. In comparison, the ^1^H-NMR analysis in DMSO-d_6_ revealed a high coverage ratio for both encapsulated compounds. The increased amount of the CB7 coverage ratio per PEDOT unit has been associated with their better solubility in DMSO-d_6_ than in D_2_O.

## 3. Results

### 3.1. Thin Film Preparation

Thin films of the polymeric materials were obtained on glass substrates by spin coating of PEDOT∙CB7-PS or PEDOT∙CB7-PR (10 mg∙mL^−1^) solutions in DMSO/H_2_O (1/1 *v*/*v*), followed by heating at 180 °C for 10 min and then cooling to ambient temperature. The obtained films were stored in a dry laboratory box at room temperature. A dry box equipped with a rubber seal was filled with a hygroscopic gel and was kept in the glove box in a nitrogen atmosphere. The technical parameters of the spin-coated films are presented in Table S1.

### 3.2. Optical Properties

Spectroscopic ellipsometry is a reflective optical technique that involves measuring the changes in light beam polarization. This is carried out by measuring the complex reflectance ratio (ρ) according to Equation (1):

(1) $$\rho =e^{i\Delta }\tan\left(\psi \right)$$

where Ψ and Δ are the ellipsometric angles. The reflectance ρ depends on the wavelength of the light and the angle of incidence as well other experimental conditions such as temperature of the sample, humidity, or pressure. It is noteworthy that the ρ can also be determined theoretically for a specific optical system by taking into account the dielectric functions of all optical phases and their thicknesses [23]. Therefore, the complex dielectric functions $\varepsilon =\varepsilon_{1}+{i\varepsilon }_{2}$, where $\varepsilon_{1}$ is real and $\varepsilon_{2}$ is the imaginary part of dielectric function, and the thickness of the layers of individual elements of the optical system must be ellipsometrically modeled.

**Figure 2:**
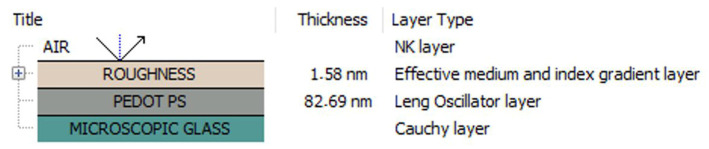
Optical model for ellipsometric measurements.

Ellipsometric measurements were performed on films deposited on opaque microscopic glass substrates using variable angle spectroscopic ellipsometry (VASE) and VTSE modes. The VASE method was used to examine the optical properties, thicknesses, and roughness of the samples at room temperature, whereas the VTSE was correlated with the DSC for the thermal transition investigation. Here, the optical system consists of four optical phases (Figure 2). Four Leng–Lorentz oscillators were used to determine the optical properties of all PEDOT films in the spectral range of 240–2500 nm.

The Leng–Lorentz oscillator layer is expressed by Equation (2) [44,45]:

(2) εE=ε∞+∑j=1NC0iE2 eiβjEgi−E−iΓjμj+e−iβjEgi+E+iΓjμj−2Ree−iβjEgi+iΓjμj−2iμjEIme−iβjEgi+iΓjμj−1+m0Ex0+ik0 

where C_0_ is the amplitude, β is the phase, μ is the order of the pole, $\omega_{g}$ is the critical frequency point, *N* is the number of oscillators, and $\Gamma_{j}$ is the broadening of oscillator. In Equation (1), E=ℏω is the photon energy, where $\hbar =6.58211\cdot 10^{-16}\;\mathrm{eV}\cdot s$ is Planck’s constant and ω is the light frequency.

The dielectric function for the surface roughness layer is approximated by the effective medium approximation (EMA) using the Bruggeman model for a material consisting of two components (PEDOT and air) [44] and calculated according to Equation (3):

(3) $$0=\sum_{i=1}^{2}f_{i}\frac{{\tilde{n}}_{i}^{2}-{\tilde{n}}_{e}^{2}}{{\tilde{n}}_{i}^{2}+2{\tilde{n}}_{e}^{2}}$$

where n_e_ is the complex refractive index of the effective medium, n_i_ is the refractive index of the inclusions, and f_i_ is the volume fractions of inclusions.

**Figure 3:**
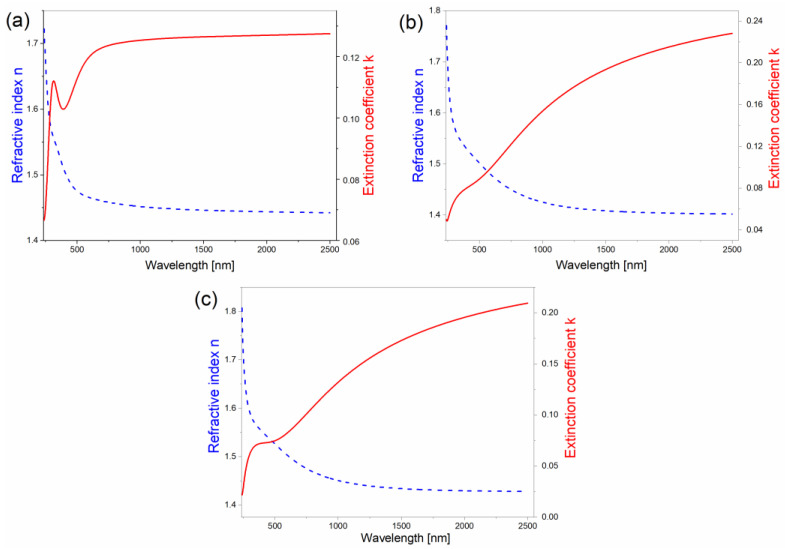
Refractive indexes and extinction coefficients of PEDOT (**a**), PEDOT∙CB7-PS (**b**), and PEDOT∙CB7-PR (**c**) as functions of wavelength.

In the first stage, the ellipsometric angles Ψ and Δ were matched to clean glass substrates, and in the second stage, they were matched to the PEDOT optical layers. The values of the optical coefficients (i.e., the refractive index n and extinction coefficient k obtained for the PEDOT, PEDOT∙CB7-PS, and PEDOT∙CB7-PR thin films at λ = 2500 nm) are presented in Table 1, and their spectral dispersions are shown in Figure 3.

The refractive index values of PEDOT, PEDOT∙CB7-PS, and PEDOT∙CB7-PR are equal to 1.44, 1.40, and 1.43, respectively. The dispersion curves obtained for PEDOT∙CB7-PR are an intermediate state between the dispersion curves obtained for PEDOT and PEDOT∙CB7-PS. Differences in the shapes of the dispersions curves are due to their molecular structures. PEDOT contains bulky pyrene ends, whereas PEDOT∙CB7-PS contains the macrocycle CB7 and no bulky pyrene ends [46]. PEDOT∙CB7-PR includes both. Table 2 presents the thicknesses of the layers and their roughness obtained using ellipsometric modeling (Figure 2) with the Spectra Ray 3 software [44].

### 3.3. Thermal Analysis

**Figure 4:**
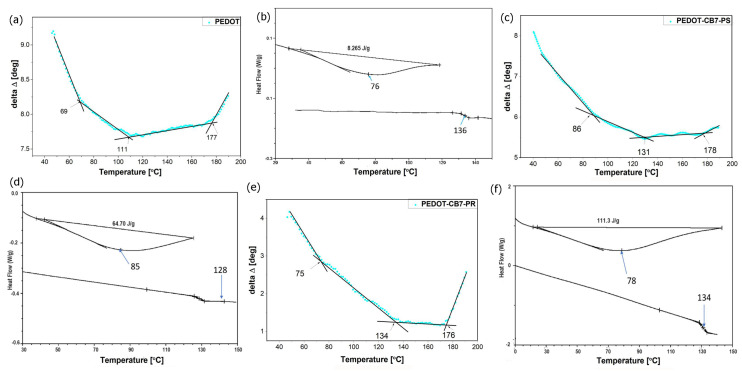
Ellipsometric angle Δ at λ = 930 nm as a function of temperature and DSC plots for PEDOT (**a**,**b**), PEDOT∙CB7-PS (**c**,**d**), and PEDOT∙CB7-PR (**e**,**f**).

Thermal analysis was performed by combining the VTSE and DSC methods. The values of the ellipsometric angles as functions of variable sample temperature were recorded for the PEDOT, PEDOT∙CB7-PS, and PEDOT∙ CB7-PR thin layers, whereas the DSC investigations were performed on their powders (Figures S1–S3). Our previous works presented a method for determining the thermal transition temperatures based on raw ellipsometric and scanning calorimetric data and a method for constructing comparison charts using both of these data [24,25]. Here, we also present the dependences of the ellipsometric angle (Δ) measured at 930 nm wavelength as a function of temperature Δ(T). We selected this value of wavelength owing to the lowest dispersion of ~200 taken points. The value of the characteristic temperature of thermal transitions was measured using the intersections of straight lines fitted one by one to groups of points. The Δ(T) dependences were generated by the special script created by our team using Spectra Ray 3 software. Each individual measurement was taken one by one at 10 s intervals with decreasing temperature values. Figure 4 shows the Δ(T) dependences obtained in the temperature range of 40–190 °C.

The trend of the obtained ellipsometric curves as a function of temperature seems to be similar for both PEDOT and PEDOT∙CB7-PS, whereas for PEDOT∙CB7-PR, the curve decreases in the temperature range of 134–176 °C. For pristine PEDOT (Figure 4a), three characteristic temperatures are visible at 69, 111, and 177 °C, respectively. The DSC plot created for the powder pristine PEDOT indicates two characteristic temperatures around 76 and 136 °C according to DSC measurements (Figure 4b). The temperatures at 111 °C and 177 °C are associated with the glass transition (*T_g_*) and the cold crystallization temperature (*T_cc_*), respectively. The endothermic peak around 69 °C is assigned to water vaporization. The ellipsometric scan of PEDOT∙CB7-PS (Figure 4c) shows three characteristic temperatures at around 86, 131, and 178 °C. The DSC plot created for the powdered PEDOT∙CB7-PS shows two characteristic temperatures at 85 and 128 °C (Figure 4d). The endothermic peak from 85 °C can be ascribed to the vaporization of the water from inside the CB7 cavities. The temperatures at 128 and 178 °C are associated with the *T_g_* and *T_cc_*, respectively. The ellipsometric scan of PEDOT∙CB7-PR (Figure 4e) also exhibited three characteristic temperatures identified at around 75, 134, and 176 °C. The endothermic peak at 75 °C originates from endothermal transition, and *T_g_* and *T_cc_* occur at 134 and 176 °C, respectively. The corresponding DSC plot appeared at 78 and 134 °C (Figure 4f).

**Figure 5:**
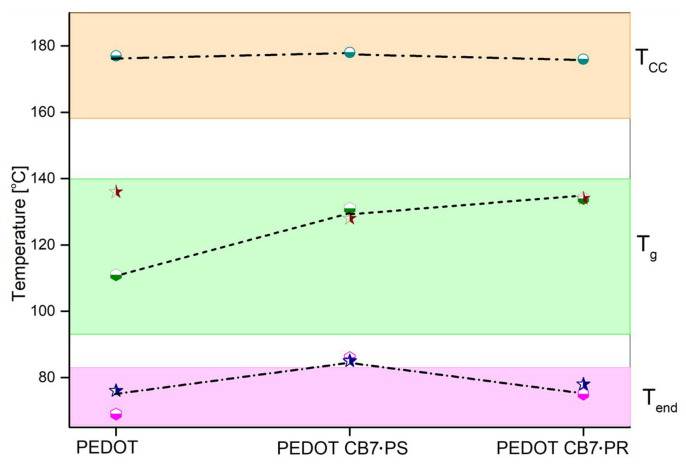
Phase diagram constructed based on DSC and VTSE results.

Further insight is provided by the construction of a phase diagram, like in our earlier works [23,24,25,47], by combining the DSC and VTSE results (Figure 5).

For the investigated PEDOT, PEDOT∙CB7-PS, and PEDOT∙CB7-PR thin films and powders, three characteristic temperatures are visible in the phase diagram. The temperatures in the pink area are the values of the endothermic peak minima associated with water evaporation. This is not a phase transition but was included in the diagram owing to the fairly high agreement of the results obtained by DSC (circle symbol) and VTSE (star symbol) analyses, giving the information when the solvent evaporation takes place. According to the high coverage ratio [21,22], the PEDOT∙CB7-PS exhibits the highest endothermic peak value at around 85 °C, thereby confirming the increased amount of water inside the CB7 cavities. The temperatures presented in the green area are constructed from the glass transition temperatures of the investigated PEDOT compounds. The *T_g_* measurements, recorded using the VTSE technique, showed an increasing trend owing to differences in the molecular weights of the investigated compounds (Figure 5). In addition, there is a visible difference between the *T_g_* of PEDOT materials using VTSE and DSC results, which may also correspond to their different investigated forms (films or powders). However, the temperature difference for PEDOT∙CB7-PS and PEDOT∙CB7-PR is in good correlation with their structures. In the orange area marked on the phase diagram, the *T_cc_* is also represented. The *T_cc_* values are almost identical in all three compounds. This observation suggests that the crystallization temperature of the PEDOT samples is connected with the presence of the pristine PEDOT phase, which is consistent with the AFM and XRD data.

### 3.4. XRD Structural Analysis

**Figure 6:**
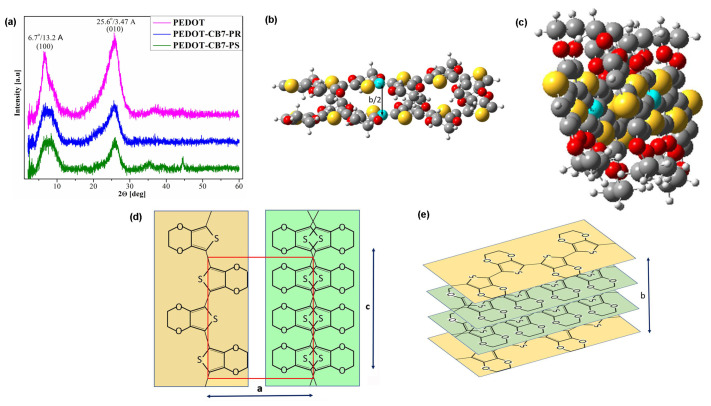
XRD analysis: XRD patterns of the PEDOT powders (**a**); optimized geometry of two PEDOT oligomers calculated with MOPAC 2016 using the PM6-DH+ semi-empirical method (**b**,**c**)—atoms: O (red), S (yellow), H (white) and C (grey); schematic arrangement of the EDOT units in the unit cell of the PEDOT crystallite (**d**,**e**), where a, b, and c are the parameters of the lattice.

Further investigations were conducted using XRD analysis. Figure 6a shows the XRD patterns of the examined PEDOT powders. Two dominant and distinct peaks at the 2Θ angles of 6.7° and 25.9° correspond to the distances between the crystallographic planes of 13.2 Å and 3.47 Å, respectively, as determined by the Bragg relation 2dsin(sinΘ) = λ. The larger interplanar distance of 13.2 Å corresponds to the ordering between the polymer chains, whereas the smaller interplanar distance of 3.47 Å can be assigned to the ordering of EDOT monomer units inside the unit cell acquired by π-π interactions [48,49,50,51]. To verify this hypothesis, geometric optimization calculations of two interacting PEDOT fragments, each containing six EDOT units, as shown in Figure 1, were performed (Figure 6b,c). Calculations were performed using MOPAC at PM6-DH^+^ [52]; i.e., PM6 with correction for the dispersion interaction and hydrogen bonding [53].

It should be noted that the distance between the marked carbon atoms belonging to different oligomers is 3.42 Å. These results support our interpretation that the dominant peak at the 2Θ angle of 25.9° on the PEDOT XRD pattern is from crystallographic planes formed by π-π stacking of aromatic rings. In addition, analysis of covalent bond lengths in the PEDOT chain shows that there is no corresponding bond length of approximately 3.4 Å. The XRD patterns of the studied powder of PEDOT compounds are consistent with a crystal structure with an orthorhombic unit cell with the lattice parameters (Figure 6a, Table S2). Our XRD results and interpretation of the PEDOT powder are in line with the data previously reported. For example, similar results were reported for PEDOT as an orthorhombic unit cell, where the lattice parameters were a = 14 Å, b = 6.8 Å, and c = 7.8 Å [54]. The clearly visible difference in the value of the lattice c parameter could correspond to the difference in the number of EDOT units that exists in the PEDOT structures. This hypothesis is clearly supported by existence of the three EDOT units in our PEDOT structures (Figure 1) compared with the two EDOT units previously reported [54]. Therefore, the investigated PEDOT crystallite unit cell contained four, whereas in our study the PEDOT contains six EDOT units. Therefore, in our case, the lattice parameter c is greater by a factor close to 6/4. Schematic arrangement of the EDOT units in the unit cell of the PEDOT crystallite is illustrated in Figure 6d,e. The values of the PEDOT crystallite structural parameters are summarized in Table S2.

### 3.5. Surface Morphology

**Figure 7:**
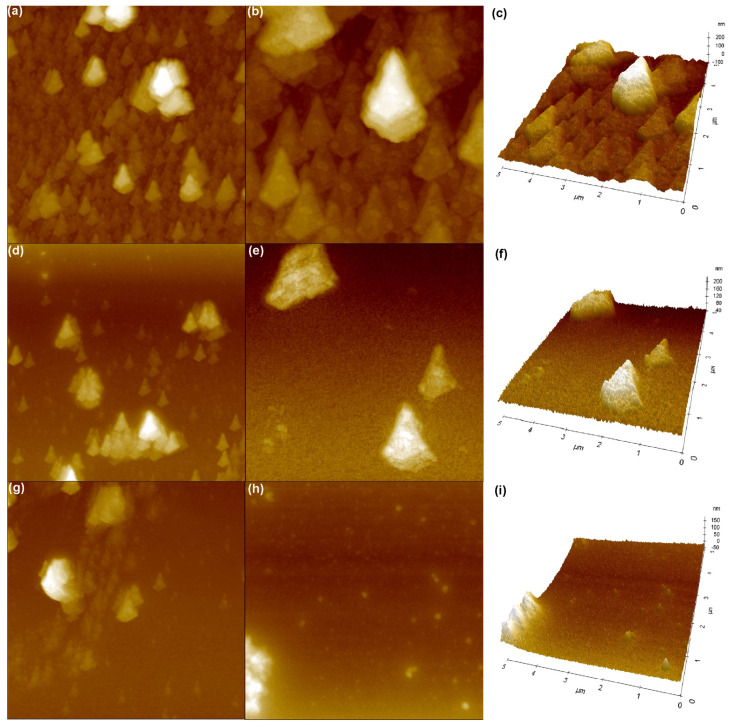
The AFM surface morphology over areas of 15 × 15 µm, 5 × 5 μm 2D, and 5 × 5 μm 3D topographic images of PEDOT (**a**–**c**), PEDOT∙CB7-PS (**d**–**f**), and PEDOT∙CB7-PR (**g**–**i**), respectively.

The AMF topographic pictures of pristine PEDOT, PEDOT∙CB7-PS, and PEDOT∙CB7-PR thin films deposited on opaque microscopic glass were further explored by atomic force microscopy (AFM) (Figure 7). The topographies of all the layers seem to be completely or partially crystallized. The root mean square roughness (R_q_) was applied for the characterization of sample surfaces. R_q_ is defined following Equation (4):

(4) $$R_{q}=\sqrt{\frac{1}{m}\sum_{i=1}^{m}{\left(Z_{i}-\bar{Z}\right)}^{2}}$$

where m is the number of points; Z_i_ is the height of every individual point; and $\bar{Z}$ is the value of their average height [55].

The high R_q_ value of the pristine PEDOT could be assigned to the increased number of crystallites. The roughness coefficient exhibits the lowest value in the case of PEDOT∙CB7-PS (Figure S4).

The AFM image of PEDOT shows conical crystals with diameters of around 1–2 μm and average heights of around 100–200 nm (Figure 7a–c). The AFM image of the PEDOT∙CB7-PS film (Figure 7d–f) indicates that the diameters of crystallites visible on its surface are similar (1–2 μm) to those of the pristine PEDOT above. It should be noted that the AFM analysis showed obvious differences on the PEDOT∙CB7-PS surface, where fewer crystallites appeared than in the PEDOT film and they occupy about 20% of the studied area. The AFM image of PEDOT∙CB7-PR exhibits the lowest number of crystallites visible on its surface (Figure 7g–i). The average diameter of the crystallites is around 0.5 μm, and they occupy only 10% of the investigated area. The AFM results are in agreement with the fact that the pristine PEDOT phase crystals contributed to the surfaces of PEDOT∙CB7-PS and PEDOT∙CB7-PR, which is consistent with the results of VTSE analysis.

## 4. Conclusions

The thermal transitions and structural characteristics of PEDOT, PEDOT∙CB7-PS, and PEDOT∙CB7-PR thin films and powders were investigated using VASE, VTSE, DSC, XRD, and AFM techniques. Ellipsometric analysis, including the determination of optical coefficients, layer thicknesses, and roughness, was performed using four optical phase systems. It turned out that the values of the refractive index for the different types of PEDOT differed slightly, and its average value was 1.42 ± 0.02. In comparison, a difference was found for the extinction coefficient value, which was equal to 0.13 for PEDOT and 0.22 ± 0.01 for PEDOT∙CB7-PS and PEDOT∙CB7-PR, proving that the complexed PEDOT exhibits slightly higher absorption. The thicknesses of the films, determined ellipsometrically, exhibited decreasing trends for PEDOT∙CB7-PS and PEDOT∙CB7-PR samples obtained from solutions with the same concentrations and conditions, confirming once again their better solubility and processability. VTSE on PEDOT films revealed the presence of three thermal transitions, while DSC revealed only two, namely, the endothermic process and glass transition in the PEDOT powders. However, the cold crystallization temperature of 176 ± 1 °C retained the same value for all investigated layers. We attribute the consistent value of the cold crystallization temperature of the PEDOT samples to the presence of the pristine PEDOT phase, which is consistent with the AFM and XRD data. Microscope analysis showed that the entire surface of the pure PEDOT film was crystallized, and conidial crystallites of similar size were visible on its surface. However, on the PEDOT∙CB7-PS and PEDOT∙CB7-PR surfaces, only single crystallites of similar sizes were visible. Using XRD diffraction, it was found that all PEDOT crystallites had an orthorhombic structure, which is consistent with the structure of the pristine PEDOT, which was confirmed by the calculation using the MOPAC 2016 program. The crystal structure favors the formation of π-π stacking of the aromatic ring system, which was also confirmed by XRD analysis. This applied approach could be extended for other supramolecular materials to build new face-on crystal orientations that are important for exploitation in various organic solar devices.

## Figures and Tables

**Table 1:** Optical constants for PEDOT material thin films at λ = 2500 nm.

	PEDOT	PEDOT∙CB7-PS	PEDOT∙CB7-PR
refractive index n	1.44	1.40	1.43
extinction coefficient k	0.13	0.23	0.21

**Table 2:** The thickness and roughness values of the polymeric thin films.

	PEDOT	PEDOT∙CB7-PS	PEDOT∙CB7-PR
Roughness [nm]	4.2	1.58	5.0
Thickness [nm]	159.4	82.7	30.0

## Data Availability

The authors confirm that the data supporting the findings of this study are available within the article and Supplementary Materials.

## Supplementary Materials

The following supporting information can be downloaded at: https://www.mdpi.com/article/10.3390/ma17061318/s1. Table S1: Summary of spin-coating parameters; Table S2: The values of PEDOT crystallite structural parameters; Figure S1: DSC plot of PEDOT; Figure S2: DSC plot of PEDOT CB7-PS; Figure S3: DSC plot of PEDOT CB7-PR; Figure S4: The mean square root of the roughness of the investigated samples.
