# Supplementary material for: Thermal Transitions and Structural Characteristics of Poly(3,4-ethylenedioxythiophene/cucurbit[7]uril) Polypseudorotaxane and Polyrotaxane Thin Films
Source: Materials (Basel). 2024 Mar 13;17(6):1318. doi: 10.3390/ma17061318 (PMC10971914; doi:10.3390/ma17061318)
Supplement: Supplementary file 1 [file materials-17-01318-s001.zip › materials-2904851-supplementary.pdf]

Supplementary Material

Thermal Transitions and Structural Characteristics of Poly(3,4-ethylenedioxythiophene/cucurbit[7]uril) Polypseudorotaxane and Polyrotaxane Thin Films

Barbara Hajduk [1,*], Paweł Jarka [2], Henryk Bednarski [1], Henryk Janeczek [1], Pallavi Kumari [1] and Aurica Farcas [3,*]

[1] Centre of Polymer and Carbon Materials, Polish Academy of Sciences, 34 Marie Curie-Skłodowska str., 41-819 Zabrze, Poland
[2] Department of Engineering Materials and Biomaterials, Silesian University of Technology, 18a Konarskiego str., 41-100 Gliwice, Poland
[3] "Petru Poni" Institute of Macromolecular Chemistry, Romanian Academy, Grigore Ghica Voda Alley, 41A, 700487 Iasi, Romania
* Correspondence: bhajduk@cmpw-pan.pl (B.H.); afarcas@icmpp.ro (A.F.)

1. *The technical parameters of spin coating films*

The investigated films were prepared by spin-coating and heating at 180 °C for 10 min and then cooled to ambient temperature from filtered DMSO/H_2O 1/1 v/v solutions of PEDOT·CB7 PS and PEDOT·CB7 PR compounds (10 mg·mL^{-1}).

Table S1. Summary of spin-coating parameters.

Material	Spinning rate [rpm]	Spinning time [s]	Atmosphere	Annealing Temperature [°C]
PEDOT	1000	60	air	180
PEDOT·CB7-PS				
PEDOT·CB7-PR				

2. *DSC analysis*

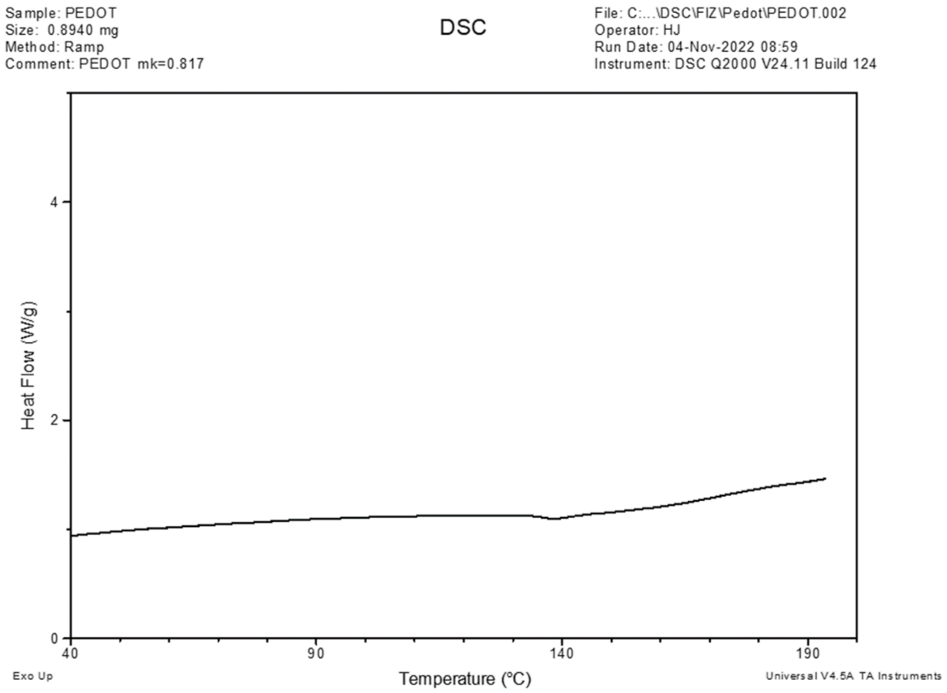

Figure S1. DSC plot of PEDOT.

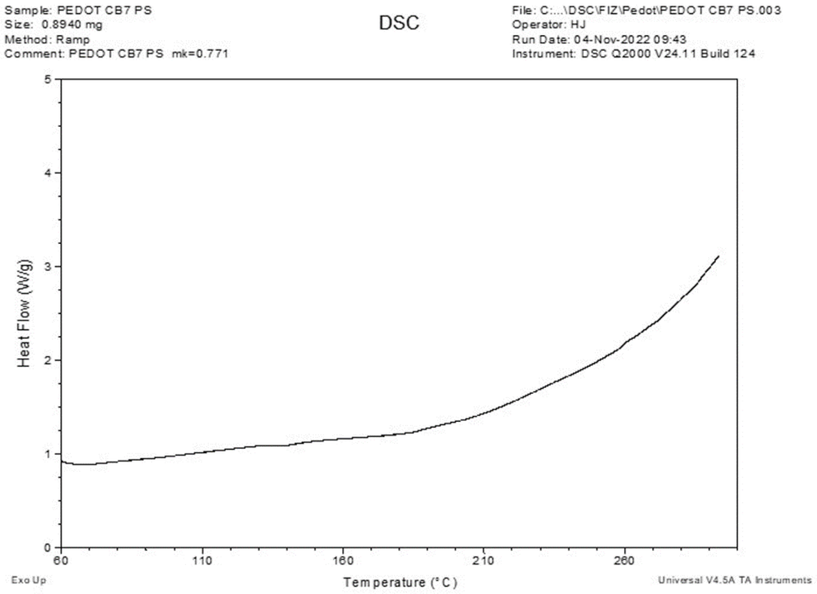

PEDOT CB7 PS. **I-run**. Heating 20°C/min. The DSC curve shows an endothermic peak at T_m=78.9°C. The vaporization of low molecular weight compounds and water in the sample. Mk=7.490

Figure S2. DSC plot of PEDOT CB7-PS.

PEDOT CB7 PR. **I-run**. Heating 20°C/min. >T=150° początki rozkładu,parowania, >T=250° rozkład?, mk=0.621mg

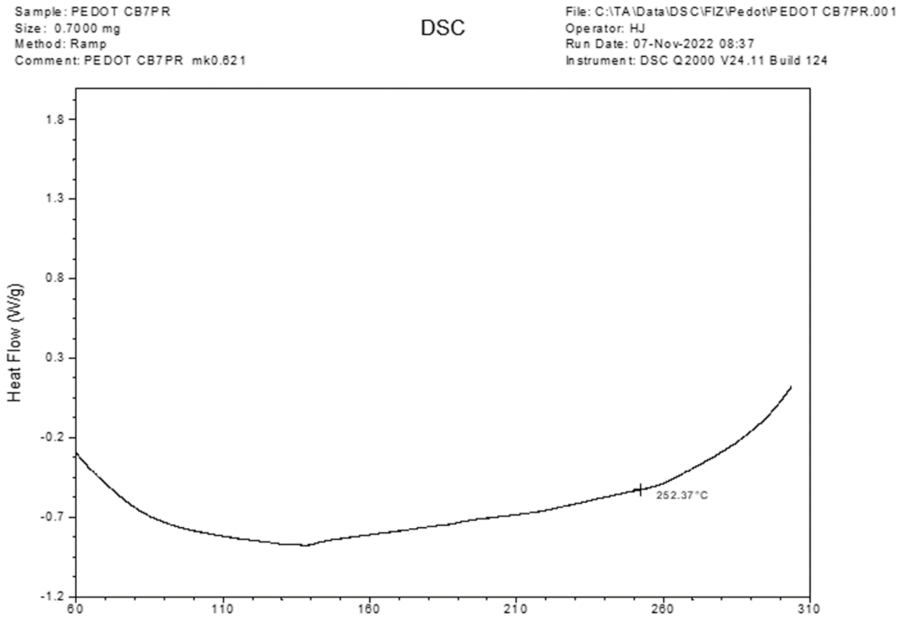

Figure S3. DSC plot of PEDOT CB7-PR.

3. *The values of PEDOT crystallites structural parameters*

Table S2. The values of PEDOT crystallites structural parameters.

Powder	Space group	Lattice parameters, ICDD, Å	Crystallite size, Å
**PEDOT**	Orthorhombic	a = 13.50<br>b = 6.70<br>c = 10.20	22.1
**PEDOT·CB7 PS**		a = 12.97<br>b = 6.80<br>c = 10.70	34.8
**PEDOT·CB7 PR**		a = 12.97<br>b = 6.81<br>c = 10.40	34.8

4. *The mean square root of the roughness of the investigated samples*

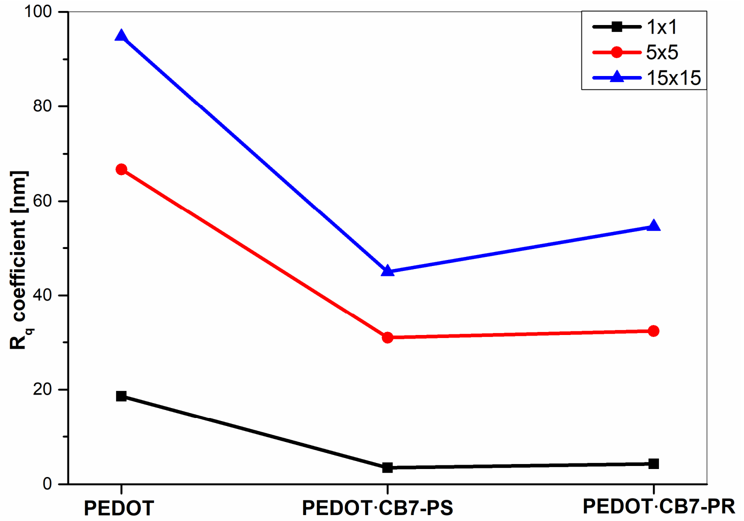

Figure S4. The mean square root of the roughness of the investigated samples.

As can be seen, the roughness coefficient has the lowest value in the case of PEDOT·CB7 PS. High *Rq* value of the pristine PEDOT could has origin from high crystallites number.
